# The B-Subdomain of the *Xenopus laevis* XFIN KRAB-AB Domain Is Responsible for Its Weaker Transcriptional Repressor Activity Compared to Human ZNF10/Kox1

**DOI:** 10.1371/journal.pone.0087609

**Published:** 2014-02-03

**Authors:** Nadine Born, Hans-Jürgen Thiesen, Peter Lorenz

**Affiliations:** Institute of Immunology, Universitätsmedizin Rostock, University of Rostock, Rostock, Germany; Hokkaido University, Japan

## Abstract

The Krüppel-associated box (KRAB) domain interacts with the nuclear hub protein TRIM28 to initiate or mediate chromatin-dependent processes like transcriptional repression, imprinting or suppression of endogenous retroviruses. The prototype KRAB domain initially identified in ZNF10/KOX1 encompasses two subdomains A and B that are found in hundreds of zinc finger transcription factors studied in human and murine genomes. Here we demonstrate for the first time transcriptional repressor activity of an amphibian KRAB domain. After sequence correction, the updated KRAB-AB domain of zinc finger protein XFIN from the frog *Xenopus laevis* was found to confer transcriptional repression in reporter assays in *Xenopus laevis* A6 kidney cells as well as in human HeLa, but not in the minnow *Pimephales promelas* fish cell line EPC. Binding of the XFIN KRAB-AB domain to human TRIM28 was demonstrated in a classical co-immunoprecipitation approach and visualized in a single-cell compartmentalization assay. XFIN-AB displayed reduced potency in repression as well as lower strength of interaction with TRIM28 compared to ZNF10 KRAB-AB. KRAB-B subdomain swapping between the two KRAB domains indicated that it was mainly the KRAB-B subdomain of XFIN that was responsible for its lower capacity in repression and binding to human TRIM28. In EPC fish cells, ZNF10 and XFIN KRAB repressor activity could be partially restored to low levels by adding exogenous human TRIM28. In contrast to XFIN, we did not find any transcriptional repression activity for the KRAB-like domain of human PRDM9 in HeLa cells. PRDM9 is thought to harbor an evolutionary older domain related to KRAB whose homologs even occur in invertebrates. Our results support the notion that functional *bona fide* KRAB domains which confer transcriptional repression and interact with TRIM28 most likely co-evolved together with TRIM28 at the beginning of tetrapode evolution.

## Introduction

Krüppel-type C2H2 zinc finger (ZNF) proteins with a N-terminal Krüppel-associated box (KRAB) form the largest family of potential transcription factors encoded in the human genome with about 400 members [1], [2]. The KRAB domain was originally described as “heptad repeat of leucines” [3]. It was deduced from human ZNF10/Kox1 transcripts isolated from a human T cell line. Independently, other researchers coined the name “KRAB” based on its occurrence together with the ZNF motifs [4]. In-depth analysis of vertebrate genomes revealed a massive expansion of ZNF and KRAB-ZNF genes during tetrapode evolution, in particular in mammals, and led to the conclusion of KRAB as a tetrapode-specific domain [1], [5]–[7]. However, the KRAB domain may have evolved from an ancestor of the histone H3 methyltransferase PRDM9/Meisetz which is also a ZNF protein. This protein appears to be the oldest gene with a KRAB-like domain since an ortholog was discovered in the sea urchin genome based on sequence similarities [8]. It was even hypothesized that homologous sequence regions from the middle of the KRAB domain can be found in all eukaryotic lineages and thus might reflect the evolutionary precursors [8]. PRDM9 plays a prominent role in meiotic recombination and in speciation (reviewed in [9]). Based on the latter and taking into consideration data on KRAB-ZNF evolution, it has been postulated that this transcription factor family might in general be important for speciation [10].

The KRAB domain is a protein-protein interaction domain. It is sufficient to confer transcriptional repression in heterologous reporter assays when tethered to a DNA binding domain [11]–[13]. This activity is dependent on the interaction of KRAB with the nuclear hub protein TRIM28 [14], [15] that was initially visualized as “silencing mediating protein 1” (SMP1) by electrophoretic mobility shift assay and co-immunoprecipitation [16]. Biochemical studies showed that a TRIM28 homotrimer likely complexes one KRAB molecule. The contact is made through the tripartite N-terminal RBCC (RING finger, B-box, coiled-coil) portion of each TRIM28 [17], [18]. Transcriptional modulation by a KRAB/TRIM28 module is thought to start with the binding of a KRAB-ZNF protein to DNA through its zinc finger motifs. The zinc finger binding specificity would therefore determine the DNA target sites. KRAB recruits TRIM28 and its associated partners that include chromatin modifying protein complexes. Histone deacetylation, histone methylation and local deposition of HP1 heterochromatin proteins are thought to result in the formation of heterochromatin and thus confer transcriptional repression [19]–[21]. The obvious assumption that KRAB-ZNF proteins act as transcriptional repressors through binding to the promoter regions of their target genes was substantiated in several studies (e.g. [22]–[25]. However, chromatin immunoprecipitation studies with antibodies against two KRAB-ZNF proteins revealed prevalent association with the transcribed regions of their target genes, particular their 3′ ends [26], [27]. Interestingly, the latter studies showed that many target genes were themselves encoding ZNF and KRAB-ZNF proteins. Furthermore, the data were consistent with the idea that KRAB-ZNF proteins do not necessarily confer transcriptional repression only, but may also transactivate or have other not yet defined roles. A more general role for silencing of genomic loci by KRAB-ZNF proteins was revealed by the participation of particular KRAB-ZNF proteins in imprinting [28]–[30] and in restricting retroviral sequences in the genome [31]. The general involvement of TRIM28 in imprinting and retroviral silencing shown in independent studies [32], [33] probably suggests that other KRAB-ZNF proteins participate in these processes as well. The numerous biological functions KRAB zinc finger proteins can exert have been recently reviewed in detail [34].

The KRAB domain can be subdivided into 2 subdomains that are usually encoded by separate exons. The KRAB-A domain is a prerequisite for efficient transcripitional repression in heterologous reporter assays and for interaction to TRIM28 [11], [13], [35], [36]. Numerous KRAB-ZNF proteins actually carry only this subdomain alone [1]. The second subdomain is called KRAB-B and exists as different kinds called B (“capital B”), b (“small b”), BL (B, long form) and C [1], [4], [37], [38]. In KRAB domains that contain them, a KRAB-B (“capital B”) type subdomain is necessary for potent repression activity and interaction with TRIM28 while the b subtype is dispensible for repression and TRIM28 association [18], [35], [36]. The C subtype does not potentiate the repression activity either, but apparently can improve interaction with TRIM28 [37], and the long B variant has not been investigated yet. The reason for these differences remains unknown and the number of studies investigating functional KRAB subdomain differences are so far limited.

XFIN, a *Xenopus laevis* protein containing a remarkable large number of 37 C2H2 zinc finger motifs [39] was early on recognized to contain a KRAB domain [4]. It was described to be expressed throughout embryogenesis and also in some adult tissues in a cell type-specific manner [40]. The same study reported cytoplasmic localization of the XFIN protein. Interestingly, the protein appears to have higher affinity towards RNA compared to DNA [41]. However, with the exception of these early reports, XFIN's function and cell biology have not been further investigated. Likewise, it was not examined if the KRAB domain of XFIN can confer transcriptional repressor activity. Because of their amphibian origin and thus as derivatives from the most distant tetrapode class compared to mammals, XFIN sequences have been employed as outliers to root phylogenetic trees of KRAB-ZNF genes/proteins [42], [43].

In this study, we compared the properties of the XFIN and PRDM9 KRAB domains with the KRAB domain of human ZNF10/Kox1 as “gold standard”. The results showed that the corrected XFIN KRAB-AB domain displayed considerably weaker transcriptional repressor activity than ZNF10 KRAB-AB. The main reason was the weak boostering ability of XFIN KRAB-B as shown by domain swapping experiments. These differences in repression activity coincided with the different extent of interaction with TRIM28. In contrast to ZNF10 and XFIN, neither the KRAB domain-related domain of PRDM9 nor its whole N-terminal part conferred transcriptional repression in reporter assays. Our study contributes functional data on previously poorly characterized KRAB domains of ancient evolutionary origin.

## Materials and Methods

### Expression and reporter plasmids

**Table S1** lists the sequences of the oligonucleotides used in the following recombinant DNA constructions. Fragments resulting from PCR were sequenced for verification. The eukaryotic expression vector pN2-GST was constructed as follows: The coding sequences for EGFP were removed from pEGFP-N2 (Clontech) by *BamH*I/*Not*I digestion and the backbone religated with a double-stranded oligonucleotide forming appropriate *BamH*I/*Not*I overhangs. The coding sequence for glutathione S-transferase from *Schistosoma japonicum* (GST) was amplified by PCR from pGEX-6P-1 (GE Healthcare) and cloned as a *Hind*III/*Sal*I fragment into this modified pEGFP-N2 to get pN2-GST. KRAB domain encoding sequences were inserted downstream of the GST cassette using *Xho*I/*Sal*I. The respective DNA fragments were generated by PCR using templates for human ZNF10/Kox1 (Refseq NP_056209; [3]); to generate ZNF10-AB and ZNF10-A) or a mutated form of this domain (ZNF10-PP-AB; [44]). In the latter construct, sequences encoding two prolines are occupying the positions before the codon of amino acid Glu-45. The KRAB-AB domain of *Xenopus laevis* XFIN was cloned by RT-PCR from total RNA isolated from larval stage 59 day 45 (RNA kindly provided by Christof Niehrs, German Cancer Research Center, Heidelberg, Germany; see GenBank accession EU277665.1). XFIN KRAB-A was cloned by PCR from the AB-part. Fragments encoding seamless domain swaps between ZNF10-AB and XFIN-AB domains (ZNF10-A-XFIN-B and XFIN-A-ZNF10-B) were obtained synthetically through a commercial service (Mr. Gene GmbH, Regensburg, Germany). In parallel, the KRAB-encoding sequences were inserted as *Xho*I/*Sal*I fragments into the pM3 effector plasmid. pM3 is an eukaryotic expression vector encoding N-terminally the DNA binding domain of the yeast transcription factor Gal4 ([45]) for use in heterologous reporter assays. In addition, these constructs were further modified to encode the strong nuclear export sequence (NES) of the human cAMP-dependent protein kinase inhibitor alpha (PKIalpha; sequence NSNELALKLAGLDINKTE; [46]). A double stranded oligonucleotide with 5′ overhangs that encoded this NES was inserted into the *Sma*I/*Bam*HI sites sitting between the sequences encoding the Gal4 and the KRAB domains. The coding sequence for the N-terminal half of human PRDM9 (Refseq NP_064612) was cloned by RT-PCR from human testis RNA (Clontech) and different fragments were generated by PCR and also inserted by *Xho*I/*Sal*I into pM3. The expression vector for human TRIM28, pCMV-TIF1beta-flag, was kindly provided by Walter Schaffner, University of Zürich, Switzerland ([47]). Luciferase reporter constructs were based on commercial plasmids. The luciferase reporter plasmid pGL2control-(5′Gal4)_5_ originated from pGL2control (Promega). It was modified by insertion of five DNA binding sites for the yeast transcription factor Gal4 DNA-binding domain into the *Bgl*II site upstream of the strong SV40 viral promoter. The *Renilla* luciferase plasmid pRL-TK was obtained from Promega.

### Cell culture, transfection and reporter assays

The adherent cell lines were cultivated in standard tissue culture plasticware (Greiner). Human epitheloid cervix carcinoma cell line HeLa (obtained from the German Cancer Research Center in Heidelberg, Germany) was grown in DMEM, 10% fetal calf serum and antibiotics at 37° and 5% CO2. *Xenopus laevis* A6 kidney cells (American Type Culture Collection CCL-102 [48] and *Xenopus laevis* XTC-2 fibroblast cells [49] (both kind gifts from Ulrich Scheer, University of Würzburg, Germany) were cultivated in 55% (v/v) Leibovitz L15 medium (Gibco), 35% sterile distilled water or 65% (v/v) L15 medium, 25% sterile distilled water, respectively, supplemented with 10% fetal calf serum and antibiotics at room temperature. The ray-finned fish cell line EPC (American Type Culture Collection CRL-2872, originally described to be obtained from the carp *Cyprinus carpio* [50], but later on found to be from the minnow *Pimephales promelas* [51]; a gift from Edda Siegel, Department of Biosciences, University of Rostock) was kept in Leibovitz L15 medium (Gibco), supplemented with 10% fetal calf serum and antibiotics at room temparature. All cell lines were trypsinized (0.05% Trypsin-EDTA solution, Gibco) for passaging.

Transfections were done with commercial reagents according to the manufacturer's recommendations. HeLa and A6 cells were transfected with Fugene HD (Roche) using 3 µl transfection reagent/µg DNA while the EPC cell line was initially transfected with Fugene 6 (Roche; 3 µl/µg DNA) and later on with NanoJuice (Novagen; 1.5 µl core reagent and booster reagent each/µg DNA).

Heterologous reporter cell assays were performed with cells grown in 6-well plates and using firefly (pGL2control-(5′Gal4)_5_, 0.5 µg) and *Renilla* (pRL-TK, 10 ng) luciferase reporter plasmids that were transfected together with the to be tested effector plasmid (1.5 µg). The effector plasmids encode fusion proteins between the DNA binding domain of the yeast transcription factor Gal4 (abbreviated “Gal4” throughout the manuscript) and the respective KRAB domain. The Gal4-part enables binding to Gal4 upstream binding sites in the firefly reporter plasmid. Reporter activities were measured 24 hours after transfection using the dual luciferase reagent system (Promega) as specified by the manufacturer and a Berthold single channel luminometer (Lumat LB9501). Within each experiment three replicates were done for each plasmid combination. The relative luciferase light units were normalized with the *Renilla* activities for each sample and the obtained values for the replicates were averaged. Fold repression was calculated by dividing the relative normalized luciferase activities of the negative control of the Gal4 DNA-binding domain alone through the activities computed for the tested effectors.

### Protein extracts, immunoprecipitation and Western blotting

After a brief rinse with ice-cold PBS, total sodium dodecyl sulfate (SDS) - denatured protein extracts were prepared by lysing cells with Lämmli SDS sample buffer. The extracts were sheared through QiaShredder columns (Qiagen) and centrifuged for 5 min at 16,000× *g* at 4°C. Aliquots from the resulting supernatant were either directly loaded to standard 10 and 12% Lämmli-type SDS polyacrylamide gels or, for concentration, first precipitated using methanol/chloroform ([52]) and then re-dissolved in a smaller volume of sample buffer before loading. For immunoprecipitations, cells were lysed for 10 min on ice in buffer TST (20 mM TRIS/HCl pH7.5; 60 mM KCl, 15 mM NaCl, 10 mM MgCl_2_, 1 mM CaCl_2_, 250 mM Sucrose, 0.5% Triton X-100), freshly supplemented with 1 mM DTT, Complete® EDTA-free protease inhibitors (Roche) and 1 mM sodium orthovanadate, 40 mM beta-glycerophosphate phosphatase inhibitors. Extracts were passed through QiaShredder colums and were cleared by centrifugation at 16,000 *g* for 10 min at 4°C. Protein concentrations in the resulting supernatants were measured by Bradford reagent (BioRad; using bovine serum albumin as standard). Immunoprecipitation (input of 1.25 mg total protein for each sample) was performed as described previously [53] using protein-G agarose beads (Roche) and rabbit polyclonal antibodies against GST (3 µg IgG per sample; Santa-Cruz Biotechnology sc-459). The SDS sample buffer eluates of the beads were loaded on the Lämmli SDS gels.

After electrophoresis, the separated proteins were electroblotted to low-fluorescence PVDF membranes (Immobilon-FL, Millipore) by semidry electroblotting (2 h at 25 V using BioRAD electroblotter) and immunostaining was carried out with primary antibodies and fluorescently labeled secondary antibodies at room temperature. Membranes were stained with 0.3% (w/v) Ponceau S in 3% (w/v) trifluoroacetic acid to control for even transfer. In order to avoid overbearing signals from reaction of secondary antibodies against the heavy chains of the antibodies used for immunoprecipitation, the blots were cut horizontally into different molecular weight ranges that could be processed individually. Blots were then destained in distilled water, blocked with blocking buffer (proprietory blocking buffer from LI-COR, diluted 1∶2 with PBS) overnight, and then probed with a respective mixture of primary antibodies (mouse monoclonal antibodies: anti-TRIM28, BD Biosciences 610681, used at 0.125 µg/ml; anti-GAPDH, Abcam ab8245, used at 0.1 µg/ml; anti-Gal4, Santa Cruz Biotechnology sc-510, at 0.2 µg/ml; rabbit polyclonal antibodies: anti-GST Santa-Cruz Biotechnology sc-459, used at 0.2 µg/ml; anti-Gal4, Santa Cruz Biotechnology sc-577 at 0.2 µg/ml) for 2 hours and secondary antibodies for 1 hour (goat anti-rabbit IgG or goat anti-mouse IgG conjugated to either IRDye800CW or IRDye680CW; LI-COR; both used at 1∶10,000), both in blocking buffer supplemented with 0.1% (w/o) Tween 20. After each antibody incubation the blots were washed 4 times 5 min each with PBS, 0.1% (w/v) Tween 20. Fluorescence signals from the blots were visualized using the Odyssey® fluorescence imager (LI-COR). Signals from IRDye800CW and IRDye680CW dyes were recorded in the green and the red channels, respectively. For representations in the figures, the original 16-bit greyscale images were loaded into Adobe Photoshop CS3 v10.0.1, and sequentially subjected to the adjustment commands “auto-contrast” and “invert” and then reduced to 8-bit. Whole blots were cropped to save space in the figures. The original 16-bit scans of the Western blots were loaded into the Odyssey application software 3.0.16 (LI-COR) and individual bands selected by manual feature selection with the rectangle tool. Signals were quantified as integrated intensity values that were corrected for local median background.

### Immunofluorescence microscopy and export assay

Cells grown on glass coverslips were fixed in PBS 4%/w/v) paraformaldehyde, permeabilized in PBS, 0.5% (w/v) Triton X-100 and subsequently stained sequentially with primary and secondary antibodies as described in detail previously [54]. In case of the EPC fish cells, coverslips were coated with 0.01% poly-L-lysine for 15 min before seeding. Primary antibodies were: rabbit polyclonal anti-Gal4 (Santa Cruz Biotechnology sc-577 at 2 µg/ml), mouse monoclonal anti-Gal4 (Santa Cruz Biotechnology sc-510, 2 µg/ml), mouse monoclonal anti-TRIM28/TIF1beta (BD Biosciences #610680 at 0.625 µg/ml). Secondary antibodies were all from goat: anti-rabbit IgG conjugated to Fluoprobes-488 (Interchem FP-GARBTTGY488; used at 2.5 µg/ml), anti-mouse IgG conjugated to AlexaFluor-488 (Invitrogen A-11001; used at 4 µg/ml) or Cy5 (Jackson ImmunoResearch 115-175-146; 3.75 µg/ml). Images were acquired with a confocal microscope (Leica TCS2 AOBS) using an 63× oil immersion lens (numerical aperture of 1.32). The image panes of the figures were processed with Adobe Photoshop CS3 v10.0.1 (german version). If necessary for clearer illustration, brightness and contrast were carefully adjusted. However, adjustments were then always performed for the whole pane in a way that did not distort or change original features.

Nuclear export of Gal4 DNA binding domain fusion proteins with NES and the specified KRAB domain was assessed microscopically 24 (HeLa cells) or 48 (A6, EPC cells) hours after transfection after indirect immunofluorescence staining. In each independent experiment at least 100 cells per coverslip were inspected and manually scored for cells with nuclear signals brighter than cytoplasmic ones (Nuc>Cyt), about equal signal distribution (Nuc≈Cyt) or with the cytoplasmic signals being the stronger ones (Cyt>Nuc). Gal4-staining in HeLa and EPC cells was done with the polyclonal antibody from rabbit, whereas in A6 cells we had to rely on the monoclonal anti-Gal4 antibody since the one from rabbit displayed unacceptable high background in the nucleus for correct scoring.

### Bioinformatics

**Figure 1:**
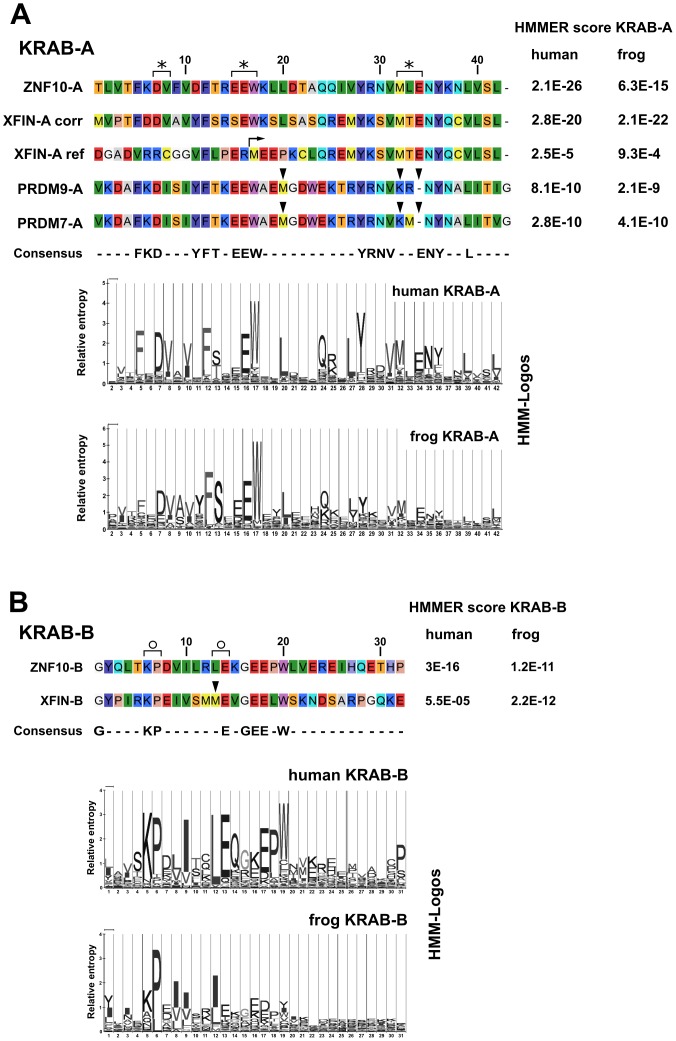
Comparative depiction of the KRAB domain sequences of ZNF10, XFIN, PRDM7 and PRDM9. Alignment of KRAB-A (**A**) or KRAB-B (**B**) subdomains and comparison to the respective human and frog HMM models. The sequences were derived from NCBI Refseq database entries for human ZNF10/Kox1 (ZNF10; NP_056209), *Xenopus laevis* XFIN (XFIN-ref; NP_001095247), human PRDM7 (NP_001091643) and human PRDM9 (NP_064612). The corrected XFIN-A sequences were derived by in-silico translation from GenBank EU277665 (labeled XFIN-A corr; see text for details). Brackets with an asterisk denote amino acid groups whose mutation have been shown to disrupt transcriptional repression, whereas those with open circle denote positions where mutation had not much effect [11]. Arrowheads point to amino acids that might be responsible for observed functional differences (see text). The right arrow marks the methionine that has been considered the start of the XFIN protein in the database reference sequence. The consensus reflects amino acids in at least 60% of the molecules at each position. HMMER scores against respective human or *Xenopus* HMM matrices are given to the right of each sequence. The HMM matrices are visualized as HMM-Logos [57] at the bottom of each sub-figure. Note, that the amino acid positions in the logo are aligned with the ones in the sequence alignments.

Amino acid sequence alignments were made with CLC Main Workbench 6.7.2 (proprietory algorithm). The alignments of the KRAB-A and KRAB-B subdomains are based on the respective amino acid sequences of ZNF10 as guide that are encoded by the KRAB-A and -B exons. KRAB domains were scored with profile hidden Markov models (HMM) of human KRAB-A and -B HMM matrices [1] or *Xenopus* ones using the “hmmpfam” subprogram of HMMER 2.3.1 [55]. Lower “expectation value scores” (E-values) indicate higher concordance with the model. Note that the E-value is dependent on sequence length of a HMM model. For high sensitivity, the E-value threshold was used at 0.01. The amphibian HMMs were computed using the “hmmbuild” subprogram of HMMER based on a multi-sequence alignment of amphibian KRAB domain containing proteins with ClustalW. Amphibian KRAB domain sequences were compiled from ENSEMBL (*Xenopus tropicalis* genome release JGI_4.2) using the strategy described in detail by others [56] and from BLASTp searches with the XFIN-KRAB-AB (our corrected sequence) against NCBI amphibian sequences (taxid:8292, amphibians; March 2013). Obvious duplicates were manually removed. Initial domain assignments were done using the human HMM matrices of the KRAB domains. To increase the sensitivity of the detection of potential KRAB-B domains in amphibians, the first-round amphibian HMM build was used to re-screen the amphibian KRAB domain sequences. The resulting hits were then used to build the second-round amphibian HMM for KRAB-B which is visualized in **Figure 1B**. All sequences were from the genus *Xenopus* and are listed in **Table S2**. HMM matrices of the respective KRAB-A and -B subdomains were visualized as HMM-Logos [57]. Different packages of the NCBI BLAST webservice interface (http://blast.ncbi.nlm.nih.gov) were used to look for orthologs of TRIM28 in different species as indicated in the text. The lungfish sequences were kindly provided by Chris Amemiya (Benaroya Research Institute, Seattle, U.S.A.), who did tblastn searches using the putative coelacanth TRIM28 ortholog against his local cumulative lungfish transcript database of five tissues obtained through next generation RNA sequencing [58].

## Results

### The corrected N-terminus of *Xenopus laevis* XFIN constitutes a *bona fide* KRAB domain with transcriptional repression potential

Transcriptional repressor activities of the KRAB-AB domain of XFIN were studied in a classical heterologous luciferase reporter assay. As a derivative of the frog *Xenopus laevis*, this protein represents a member of the KRAB zinc finger protein family from the evolutionary oldest class of tetrapodes. We first cloned the XFIN KRAB domain using primers that were derived from the reference database sequence. Since the predicted N-terminus of XFIN in the database would comprise a N-terminally truncated KRAB-A subdomain we choose to extend the sequence based on the alignment to the first described member of the KRAB domain family, human ZNF10/Kox1 (see **Figure 1A**). When we controlled the KRAB-AB domain of XFIN which we obtained after RT-PCR from *Xenopus laevis* larval stage 59 RNA by sequencing, we noticed an additional deoxycytidine insertion in all clones (registered in GenBank as EU277665). Surprisingly, in-silico translation of this cloned sequence resulted in a KRAB-A amino acid sequence that aligned much better to ZNF10-A (**Figure 1A**). Sequence comparisons to KRAB-A sequences from human and frog using profile hidden Markov models (HMM) corroborated that the cloned XFIN KRAB-A domain fit much better to the KRAB-A model and the consensus sequence (E-values improved for both, human and frog HMMs, see **Figure 1A**). In particular two residues, ^7^D^8^V (marked with asterisk in **Figure 1A**), that have been shown to be important for KRAB function [11], [13], are now conserved in the corrected sequence. The second half of KRAB-A (starting from ^24^Q) and KRAB-B are identical in the former reference and the corrected XFIN KRAB domain. The XFIN KRAB-B subdomain appeared to be a relatively distant one compared to the human ZNF10-KRAB-B when scored against the human subdomain HMM (relatively high E value of 5.5×10^−5^ versus 3×10^−16^, **Figure 1B**). When referred to the frog HMM, however, the KRAB-B subdomains scored slightly better (**Figure 1B**). To independently confirm the sequence findings we then cloned XFIN-AB sequences from two *Xenopus laevis* cell lines, A6 and XTC-2 by RT-PCR. Again, all clones contained the same deoxycytidine insertion (see GenBank accessions EU277666 and EU277667) that resulted in the altered open-reading frame at the N-terminus. Thus, our data support a corrected version of the N-terminal part of the XFIN protein with a conserved KRAB-AB domain.

**Figure 2:**
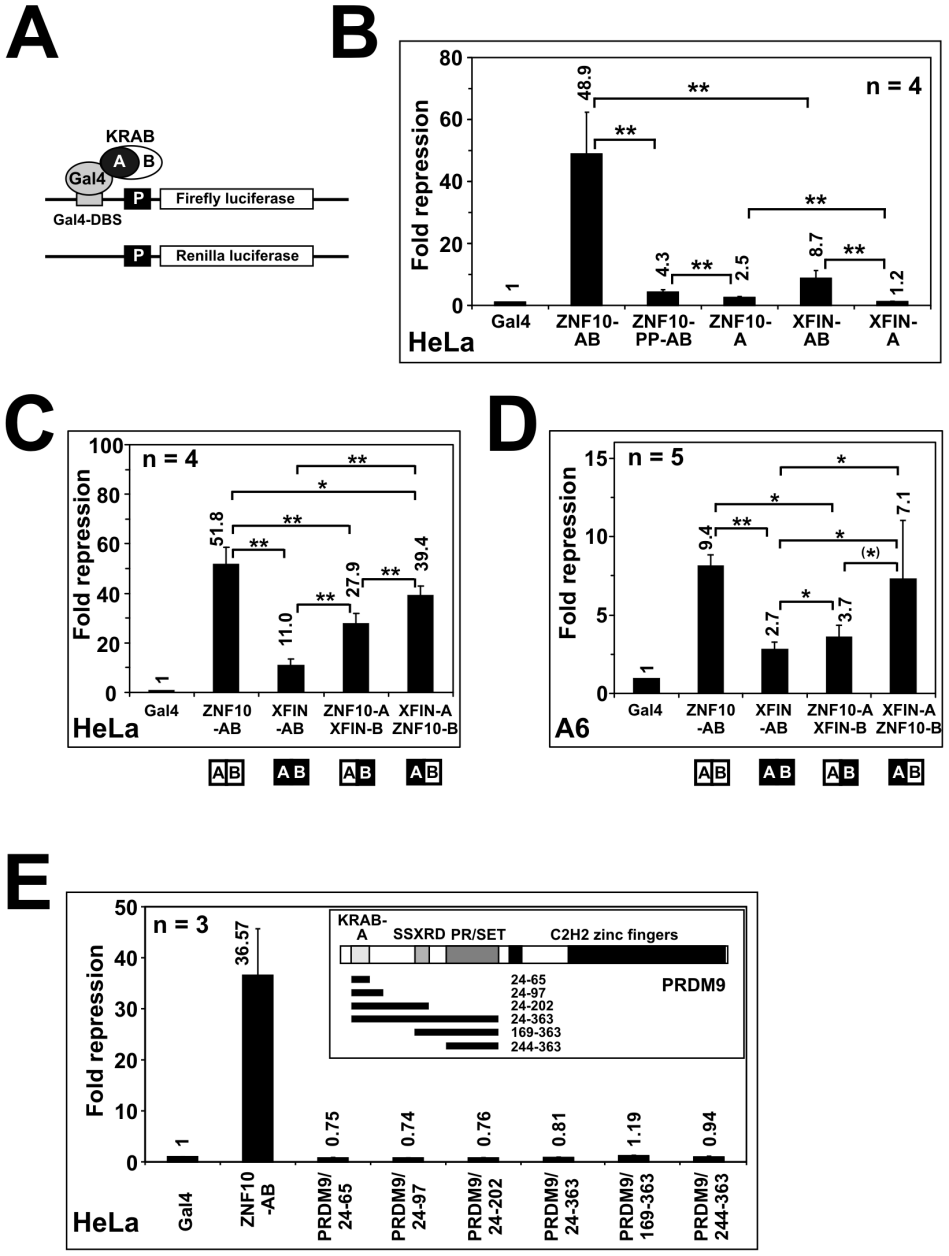
Evaluation of the transcriptional repression potential of different KRAB domains. Heterologous luciferase reporter assays using fusions between the indicated KRAB domains and the Gal4-DNA-binding domain (Gal4). Results of 3–5 independend experiments (n = 3–5, see charts). Asterisks denote statistical significance in a two-tailed paired T-test (one asterisk in brackets means p<0.055; one asterisk p<0.05, two asterisks p<0.01 and three asterisk p<0.001); **A**: Illustration of assay; only the firefly luciferase reporter carries upstream Gal4 DNA-binding sites (Gal4-DBS) while the *Renilla* luciferase does not and is used for normalization. **B**: Assay in human HeLa cells, comparing Gal4 as baseline (set to 1) with its fusions to the indicated KRAB domains/subdomains. **C**: KRAB-B domain swapping experiment in human HeLa cells, switching the ZNF10-B domain to XFIN-A and vice versa. **D**: Same experiment as C, but done in *Xenopus laevis* A6 cells. **E**: Testing of various N-terminal parts of PRDM9 in human HeLa cells, numbers designate amino acid positions in the full-length protein. PRDM9 domain abbreviations: SSXRD = SSX repression domain motif (PFAM PF09514; [78]); PR/SET = derivative of SET doman , (*Drosophila* Su(var)3–9, Enhancer-of-zeste and Trithorax; PFAM PF00856 [79].

Next, the corrected XFIN-KRAB domain was tested as fusion protein with the Gal4 DNA binding domain for transcriptional repression activity (**Figure 2**). We compared the activities of constructs expressing XFIN full KRAB-AB domain and A subdomain only, respectively, to those encoding ZNF10-KRAB-AB (positive control), ZNF10-KRAB-A and a double proline insertion mutant (ZNF10-PP-AB, known to disrupt activity; [44]) or Gal4 alone as baseline. The results in human HeLa cells indicated a clear-cut repression potential of XFIN KRAB-AB of about 9-fold (**Figure 2B**), that was considerably lower than the about 49-fold luciferase downregulation of ZNF10-AB. The difference in potency was visible as well for the isolated KRAB-A subdomains of the two proteins: While ZNF10-KRAB-A still exhibited 2.5fold repression activity, XFIN-A was inactive when compared to the Gal4 baseline. The data implied a general weaker activity of the KRAB-A subdomain of XFIN compared to that of ZNF10, as well as a weaker enhancement by the respective KRAB-B subdomain. Since all constructs were faithfully expressed at the expected molecular weight and at comparable protein expression levels, the observed differences in repressor activity can be excluded to be due to disparate protein expression (see Western blots in **Figure S1A**).

To confirm the lower extent of XFIN KRAB-B for KRAB-A potentiation we evaluated changes in transcriptional repression when KRAB-B subdomains were swapped between ZNF10 and XFIN. Compared to wildtype ZNF10-AB the ZNF10-A-XFIN-B domain chimera dropped in repression activity in Hela cells (**Figure 2C**). Vice versa, XFIN-A gained in repression potential compared to its own wildtype configuration when teamed up with ZNF10-B. The results demonstrated the importance of the KRAB-B subdomain for the general repression activity. We also assessed the same Gal4 fusion constructs in *Xenopus laevis* A6 cells (**Figure 2D**). While the absolute numbers for repression were lower for all KRAB domains, the differences between ZNF10-AB and XFIN-AB as well as the changes after the B subdomain swaps were similar to those in the human HeLa cells. Again, the ZNF10-B subdomain boostered the XFIN-A subdomain in activity more than the wildtype XFIN-B. Western blotting illustrated appropriate levels of expression for the constructs (**Figure S1B**, **Figure S1C**). Altogether the data led to the conclusion that the corrected N-terminal amino acid sequence of XFIN comprises a *bona fide* KRAB-AB domain in which a relatively weak KRAB-A subdomain is moderately enhanced in transcriptional repression activity by a likewise moderately potent KRAB-B subdomain.

### The N-terminal portion of PRDM9 does not confer transcriptional repression activity

PRDM9 and the very close relative PRDM7 are the human representatives of the *Meisetz* ortholog family which has been suggested to embody the ancestor of the KRAB domain ([8]; see introduction). Their N-terminus contains a KRAB-A box which aligns nicely with the ZNF10-A subdomain (**Figure 1A**). To assess the transcriptional repressor activity of the N-terminal portion of PRDM9, several constructs encoding various amino acid stretches of PRDM9 fused to the Gal4 DNA binding domain were used as effector plasmids in reporter assays in human HeLa cells (**Figure 2D**). Compared to the baseline Gal4 alone and the ZNF10 KRAB-AB domain, neither the PRDM9 KRAB-A nor the more extended parts including the SSX repression domain sequences evidenced transcriptional repression potential in the reporter assay. The data rather showed some minor transactivation. Constructs expressed the expected protein species with some significant deviation in expression levels for the largest constructs (see **Figure S2**). However, these differences do not invalidate our conclusions. In summary, the N-terminus of PRDM9 that includes a subdomain similar to *bone-fide* KRAB-A fails to show transcriptional repression activity in a heterologous reporter gene assay.

### XFIN-KRAB interacts less efficiently with human TRIM28 than ZNF10-KRAB due to its B-subdomain

**Figure 3:**
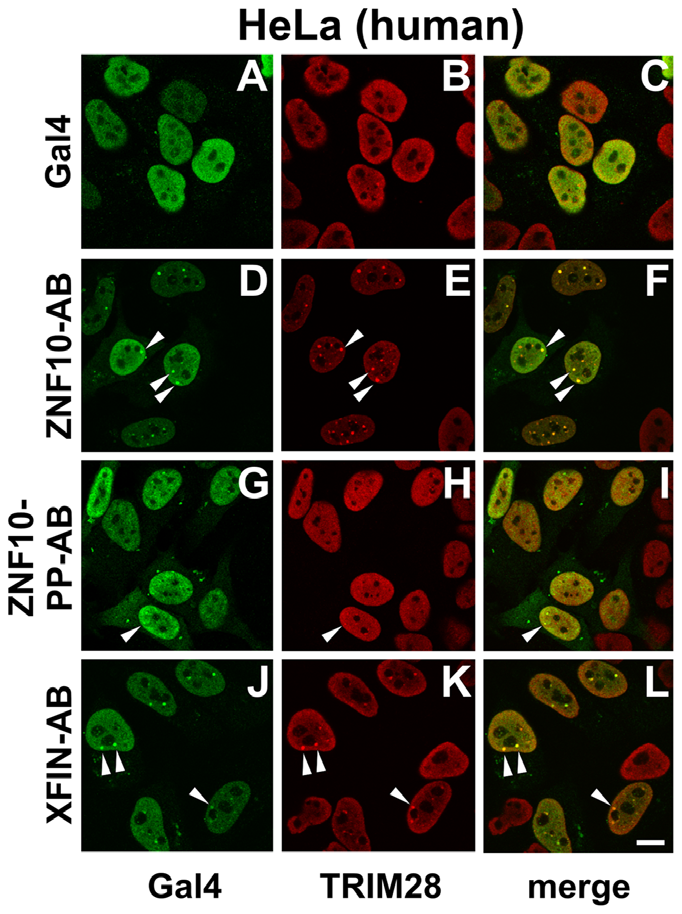
Intracellular distribution of ectopically expressed Gal4-KRAB fusion proteins and colocalization analysis with endogenous TRIM28 in human HeLa cells. 24-transfection of the indicated Gal4 effector constructs cells were fixed and stained for Gal4 (shown in green) and TRIM28 (red). Each row shows the cells of the same image pane. Arrowheads point to some of the telltale prominent foci showing accumulated Gal4 and TRIM28 proteins. Bar = 10 µm.

In general, transcriptional repression activity conferred by a KRAB-domain is thought to be mediated by the TRIM28 protein that interacts through its RBCC domain with KRAB (see introduction). In a first set of experiments we looked at the distribution of Gal4-KRAB fusion proteins in comparison to endogenous TRIM28 by immunofluorescence microscopy after transfection of human HeLa cells (**Figure 3**). Gal4 alone accumulated in the nucleoplasm. This was expected since the Gal4 DNA binding domain is known to contain a nuclear localization signal [59]. At the same time, endogenous TRIM28 was completely nucleoplasmic in a rather diffuse distribution with only occasional small aggregations. The Gal4-ZNF10-KRAB-AB protein also predominantly localized to the nucleoplasm. However, strikingly, we observed a lot of transfected cells with a few to up to more than ten small bright foci that at the same time also displayed recruitment of TRIM28. In contrast, the Gal4-fusion protein with the mutant ZNF10-PP-KRAB that is unable to act as transcriptional repressor ([44] and see reporter gene assays above) only rarely showed a few foci with TRIM28 accumulation. When Gal4-XFIN-KRAB-AB was examined, the bright foci with KRAB domain/TRIM28 colocalization were visible as well. We had the impression that the number of foci was lower compared to the ZNF10-KRAB-AB construct, but did not formally count them. Ectopically expressed KRAB-B subdomain swapped Gal4-KRAB fusion proteins exhibited similar foci formation with TRIM28 recruitment. Furthermore, the Gal4 fusion proteins with the KRAB-A subdomains of ZNF10 and XFIN formed nuclear foci colocalizing with TRIM28, too (data not shown). In the latter case, the foci also seemed less numerous than with the full KRAB-AB domain of ZNF10. The joined Gal4-KRAB/TRIM28 foci provide a first telltale indication of potential interaction of a Gal4-KRAB fusion protein with endogenous TRIM28. Consistent with the absence of repression, the Gal4-PRDM9 constructs did, in contrast, not display foci with colocalizing TRIM28 (see **Figure S3**). Gal4-KRAB domain fusions from other KRAB zinc finger proteins confirmed the presence of telltale foci and the recruitment of endogenous TRIM28 (data not shown). In further agreement, the Gal4-KRAB fusion protein foci was colocalized with cellular HP1-alpha protein, a known interaction partner of TRIM28 [20], [60] (data not shown). A similar analysis could not be done in *Xenopus* cells, since TRIM28 had not yet been described in this species and antibodies against it are not available. Yet, the existence of frog TRIM28 could be inferred from bioinformatic analyses using BLAST searches against *Xenopus* databases (see Discussion section). Interestingly, neither the ZNF10-KRAB-AB nor the XFIN-KRAB-AB Gal4 fusion protein exhibited telltale nucleoplasmic foci in frog cells (see **Figure S4A**).

While the assumption that the occurence of foci was due to interaction of the Gal4-KRAB to TRIM28 might be reasonable, colocalization does not prove physical interaction within a complex. However, if binding to endogenous nuclear TRIM28 protein would influence the localization of a Gal4-KRAB protein in an obvious manner, stable interactions might be visualized in a single cell assay. Our conceptual approach was to insert a strong nuclear export signal (NES) into all constructs immediately behind the Gal4 sequences. Provided the NES was stronger than the nuclear import signals, Gal4 should reside in the cytoplasm at equilibrium. In contrast, if a Gal4-KRAB protein interacted with endogenous nuclear TRIM28 protein, the Gal4-protein should be trapped in the nucleus, be taken out of the freely movable pool of molecules and thus display nuclear accumulation.

**Figure 4:**
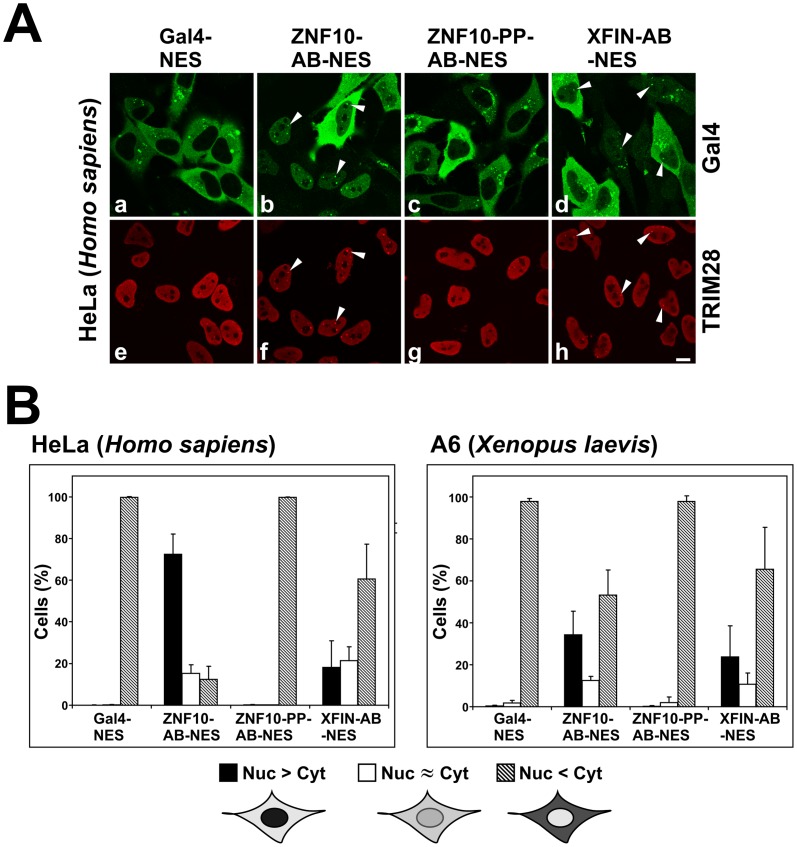
Analysis of the nuclear/cytoplasmic compartmentalization of Gal4-KRAB fusion proteins containing a strong nuclear export sequence (NES). Cells were transfected with the indicated Gal4 fusion proteins, fixed and stained for Gal4 and (in human cells) for TRIM28. Cells with Gal4 expression were manually scored under the fluorescence microscope to contain excess nuclear (Nuc>Cyt), excess cytoplasmic (Nuc<Cyt) or similar distributions (Nuc≈Cyt) and counted. **A**: Example fluorescence images from human HeLa cells (corresponding channels for Gal4 and TRIM28 staining of the same image pane one below the other). Arrowheads mark some of the foci with the telltale simultaneous Gal4-KRAB and TRIM28 accumulation. Bar = 10 µm. **B**: Quantification for human HeLa cells (7–9 independent experiments for each construct; between 134 and 442 cells counted per experiment) 24 hours post-transfection and *Xenopus laevis* A6 cells (4–7 independent experiments, between 154 and 447 cells counted) 48 hours after transfection. Two-sided T-tests looking at the data between ZNF10-KRAB-AB and XFIN-KRAB-AB Gal 4 fusions indicated p-values of p = 1.7×10^−6^ (HeLa) and p = 0.23 (A6), respectively. Exact numbers of scored cells and complete statistical analysis can be found in **Table S3**.

The results of such compartmentalization assays are summarized in **Figure 4**. First, we performed such assays in human HeLa cells. Fluorescence microscopy showed that Gal4-NES exclusively displayed cytoplasmic localization. Therefore, the prerequisite of the assay was fulfilled. In contrast, fusion of the ZNF10-KRAB-AB domain to Gal4-NES shifted the localization to a great extent into the nucleus. The telltale Gal4-KRAB/TRIM28 foci were visible again as demonstrated by co-staining with anti-TRIM28 antibodies. When looking at the ZNF10-PP-KRAB-AB mutant, the Gal4-NES fusion protein was almost exclusively cytoplasmic. For the XFIN-KRAB-AB Gal4-NES protein, localization was mostly nuclear in a minority of HeLa cells, in particular those with weaker expression of the protein. More cells showed clear-cut stronger cytoplasmic localization. Yet both, cells with mostly nuclear or cytoplasmic distribution, respectively, often displayed Gal4-KRAB/TRIM28 foci. The dependence of the results on the level of expression in individual cells was expected: Assuming the number of nuclear TRIM28 molecules was limited, saturation of respective binding sites could lead to excess free Gal4-KRAB proteins that should be easily exported. The cells were scored for the nuclear/cytoplasmic compartmentalization under the microscope to get a more quantitative evaluation (**Figure 4B**). The data showed that significantly more Gal4-NES-ZNF10-KRAB-AB fusion protein was retained in the nucleus than Gal4-NES-XFIN-KRAB-AB. However, XFIN-KRAB-AB interaction with nuclear binding sites was still obvious in the large difference to the numbers for the Gal4-NES fusion to the ZNF10-PP-KRAB mutant and Gal4-NES alone.

Next, the compartmentalization assay was done in A6 frog cells. Examples of the respective microscopical images of the Gal4-NES fusion proteins are given in **Figure S4A**. The scoring of cells from several experiments inferred nuclear retention of the ZNF10-KRAB-AB as well as XFIN-KRAB-AB Gal4-NES fusion proteins. ZNF10-KRAB-AB appears to be slightly better in that regard. However, the difference was not statistically significant, likely because of the relative large deviations between experiments. Compared to HeLa cells, the extent of nuclear retention of the ZNF10-KRAB-AB appeared to be lower in the frog cells while that of XFIN-KRAB-AB seemed to be slightly higher. It is tempting to speculate, that the human KRAB domain from ZNF10 interacts better, i.e. with higher affinity, with its authentic human TRIM28 while XFIN-KRAB might instead bind *Xenopus* TRIM28 more efficiently. The different extent of retention correlated with the transcriptional reporter assay data of ZNF10 and XFIN KRAB domains.

**Figure 5:**
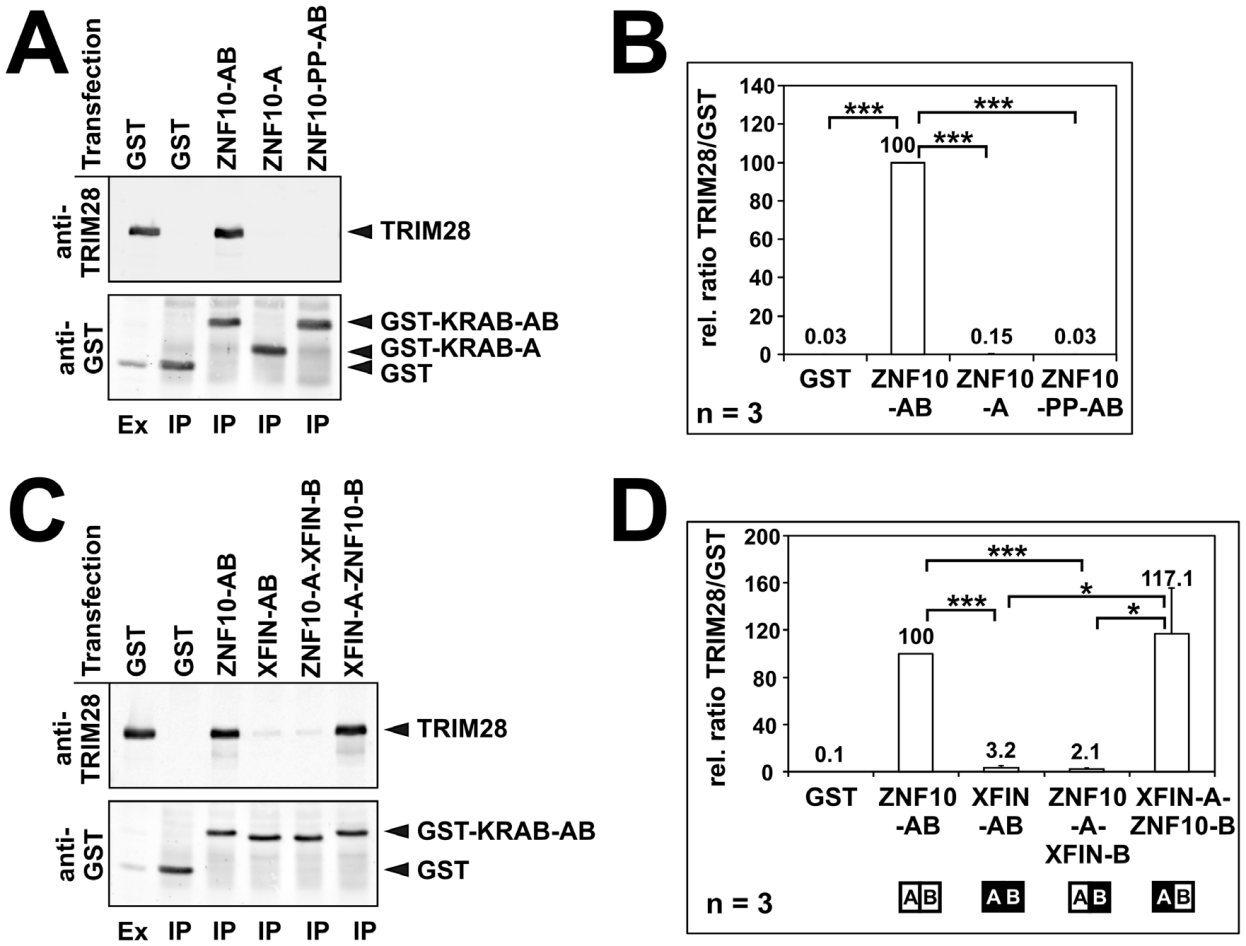
Cellular interaction analysis of different KRAB domains with the endogenous TRIM28 protein in human HeLa cells. 24-KRAB fusion constructs, extracts were analyzed by immunoprecipitation (IP) with GST antibodies followed by Western blotting to look for shared complexes with cellular TRIM28 protein. An extract sample (ex) was run as positive control for Western blot immunostaining. The upper part of the blot was probed with anti-TRIM28, the lower part with anti-GST antibodies. **A**: Example gel for the ZNF10-AB versus A only and ZNF10-PP-AB analysis. **B, D**: Quantitative evaluation of the blot signals from 3 independent experiments. Columns and numbers indicate relative TRIM28/GST-fusion ratios (ZNF-AB result set to 100%). **C**: Example gel for the comparison of ZNF10-AB, XFIN-AB and the B subdomain swaps. Statistical significance tested using a one sample 2-tailed T-test. One asterisk p<0.05, two asterisks p<0.01, three asterisks p<0.001.

Finally, we tested the stable interaction of the various KRAB domains with endogenous human TRIM28 protein in a classical co-immunoprecipitation approach. The different KRAB fusion proteins attached to GST were expressed in HeLa cells and immunoprecipitated with anti-GST antibodies. Then the eluates from the precipitation were analyzed for captured cellular TRIM28. As expected, ZNF10-KRAB-AB efficiently co-precipitated TRIM28, whereas ZNF10-KRAB-A alone or the double proline mutant of ZNF10-KRAB-AB failed to do so (**Figure 5A** **,** **Figure 5B**). When ZNF10- and XFIN-KRAB-AB domains were tested side-by-side, ZNF10-KRAB recruited endogenous TRIM28 clearly more efficiently (**Figure 5C** **,** **Figure 5D**). However, when the KRAB-B subdomains were swapped, the co-precipitation efficiency also switched, i.e. the XFIN-A-ZNF10-B subdomain fusion now captured TRIM28 to an extent comparable to ZNF10-KRAB-AB while ZNF10-A-XFIN-B dropped in performance. These findings demonstrated that the XFIN KRAB-B subdomain supports stable interaction to human TRIM28 relatively insufficiently, and that the transfer of the ZNF10 B subdomain to XFIN KRAB-A resolved this issue.

### Retention of Gal4-NES-KRAB fusion proteins and transcriptional repression activity of KRAB domains is dependent on exogenously added TRIM28 protein in fish cells

**Figure 6:**
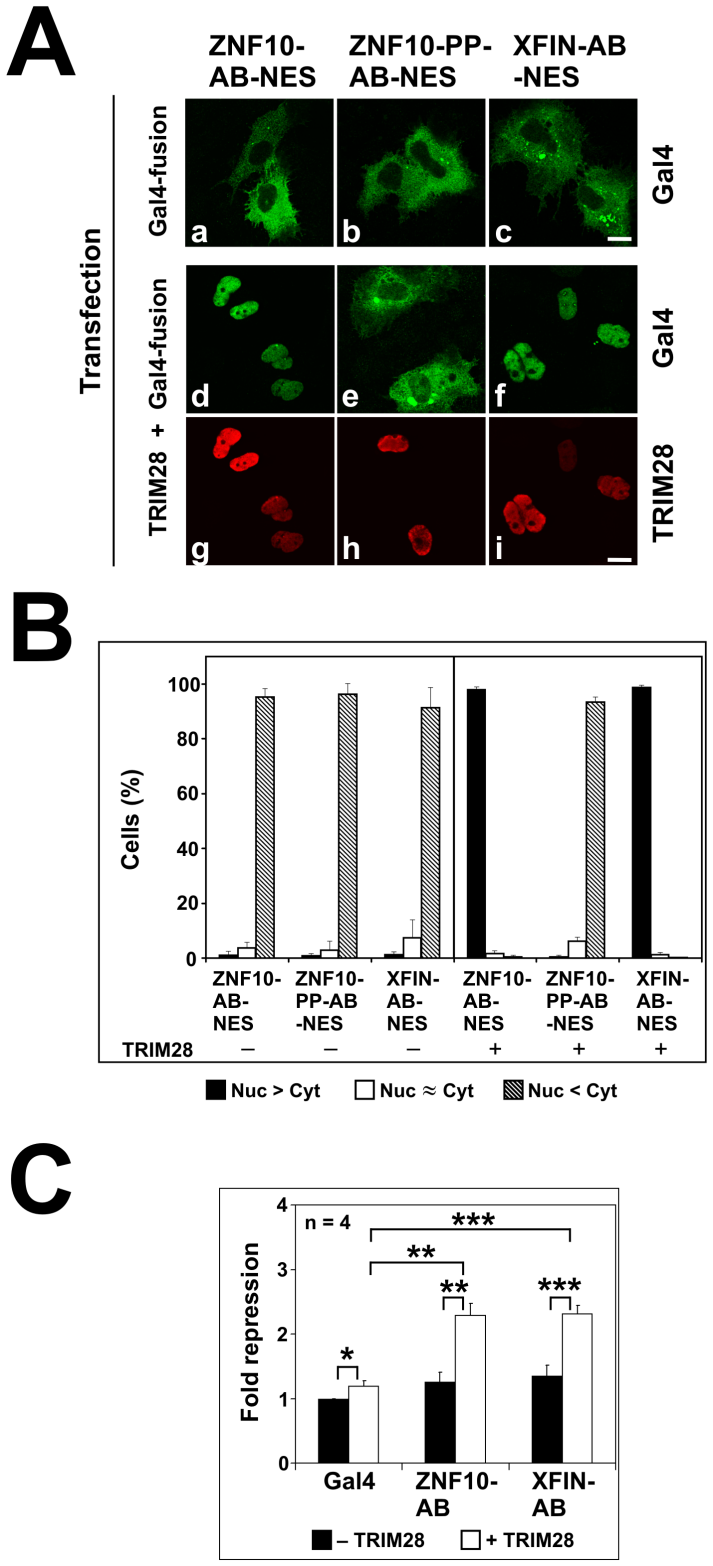
Characterization of KRAB-domains in EPC fish cells. **A**: Example of indirect immunofluorescence analysis of the nuclear/cytoplasmic compartmentalization of Gal4-KRAB-NES proteins alone (a–c, Gal4-staining) or after co-transfection with human TRIM28 (d–f, Gal4-staining; g–i, TRIM28-staining; corresponding panes one below the other). Bar = 10 µm. **B**: Quantification of the nuclear/cytoplasmic compartmentalization experiments (5–10 independent experiments for each construct, between 105 and 301 cells counted per experiment) 48 hours after transfection. Exact numbers and statistical analysis are given in **Table S3**. **C**: Transcriptional repression potential of different KRAB domains measured by heterologous luciferase reporter assays using Gal4-KRAB effector constructs as in **Figure 2**. Filled bars represent experiments without exogenous human TRIM28, open bars those with co-transfection of TRIM28. The Gal4 result of each experiment in absence of TRIM28 was used as baseline and set to 1 and four independent experiments were run. Statistical significance of a 2-tailed paired T-test is indicated by one asterisk (p<0.05), two asterisks (p<0.01) and three asterisks (p<0.001).

Vertebrate genome analysis highlights the KRAB/TRIM28 module of transcriptional regulation in tetrapode species (see introduction). In fish cells, there is no convincing example of a functioning KRAB domain protein. In ray-finned fish, a close TRIM28 ortholog does not exist either [61]. We performed compartmentalization assays and reporter assays for transcriptional repression in the *Pimephales promelas* fish cell line EPC in order to determine whether the characteristics of the tested KRAB domains are indeed tetrapode-specific. The Gal4-KRAB fusion proteins localized in the EPC cell nuclei, indicating functional import (**Figure S4B**). When assaying the compartmentalization of the Gal4-NES fusion proteins, we observed complete cytoplasmic distribution without any clear-cut nuclear retention not only for the KRAB-PP mutant of ZNF10, but also for the ZNF10-KRAB-AB and XFIN-KRAB-AB constructs (**Figure 6A** **,** **Figure 6B**). This analysis demonstrated that the latter two proteins did not encounter binding partners in the nucleus that would lead to their stable retention. After co-expression of human TRIM28 in EPC cells, however, the Gal4-NES fusions to the functional KRAB domains of ZNF10 and XFIN now virtually completely resided in the nuclei. Nuclear telltale KRAB/TRIM28 foci were not observed in fish cells. These observations provided evidence for the dependence of the KRAB proteins' nuclear retention on TRIM28 and thus their interaction with TRIM28.

The transcriptional repression potential of the ZNF10 and XFIN KRAB-AB domains were found to be negligible in EPC fish cells (**Figure 6C**). Complementation with exogenous human TRIM28 conferred significant activity, yet the extent of about 2fold repression for both domains was small. Likely, other downstream factors besides TRIM28 are missing or are not conserved enough to confer more enhanced transcriptional repressor activities. Notwithstanding, the basic machinery to confer transcriptional repression in fish cells is functional once TRIM28/KRAB complexes are formed.

## Discussion

Functional assays in frog and human cell lines demonstrated that XFIN KRAB-AB behaves like a *bona fide* KRAB domain, i.e. XFIN KRAB is sufficient to confer transcriptional repression when targeted to the promoter of a reporter cassette and is able to interact with the TRIM28 co-repressor protein. Its repressor activity was significantly lower compared to that of human ZNF10 in human and in frog cells. The reduced activity was reflected in the weaker interaction with human TRIM28 as shown by classical co-immunoprecipitation as well as by a unique single-cell nuclear export assay. The latter employs the intrinsic Gal4 nuclear localization signal and an additionally added NES. The formation of cellular Gal4-KRAB foci was taken as an initial telltale for potential KRAB/TRIM28 interaction within cells. The formation of such aggregates appeared to be dependent on a functional KRAB domain. However, an additional reason for observing aggregate formation might be due to the known dimerization property of the Gal4 DNA binding domain [62] used in the assay. The results of our nuclear export assay are mirrored in a report that was published while writing our manuscript: The authors demonstrated dependence on the KRAB domain for strong nuclear accumulation through interaction with nuclear TRIM28 using a GFP fusion protein localization assay [63]. Based on their study of a number of KRAB domains, the authors postulated a general nuclear accumulation activity of this domain and its general requirement for nuclear distribution of KRAB-ZNF proteins. While they confirmed our own unpublished data for ZNF10/Kox1, their general statement might be an overinterpretation: There are also reports that KRAB domains alone do not accumulate in the nucleus, [64], [65] and that zinc finger sequences suffice to specify nuclear localization (e.g. [65]–[67]). Indeed, some C2H2 ZNF proteins have been shown to contain non-classical nuclear localization sequences in their zinc finger domains [68]–[70]. Thus, it is likely that different KRAB-ZNF proteins will have different properties and behavior with respect to their spatial distribution and dynamics.

The higher transcriptional repressor activity of ZNF10 in human and *Xenopus* cells argues that the reason for the differences in repressor potency of the two KRAB-AB domains was not due to disparate transfection efficiencies between frog and human cells or the lack of species-specific factors. While already the XFIN KRAB-A subdomain alone was less potent, our results, in particular the swapping experiments, demonstrate that the major difference to ZNF10 was the lower boostering ability of XFIN KRAB-B for transcriptional repression as well as TRIM28 interaction. The strong KRAB-B enhancement within a KRAB-AB configuration has been initially described for ZNF10 by Vissing et al. [36], and confirmed later on [18], [35], yet its mechanisms remain elusive. Our results raised the question if the amino acid sequence might give a clue for lower boostering by XFIN-B. Regarding the amino acid residues strongly conserved in human B-domains, the most obvious difference in XFIN-B is a methionine instead of leucine in the highly conserved leucine-glutamate residue pair (see **Figure 1B**, arrowhead). In other frog KRAB-B sequences, the same position usually is taken by isoleucine or leucine (see frog HMM-Logo in **Figure 1B**). Yet, mutation of the leucine-glutamate residue pair to double alanine did not significantly change the repression potential of ZNF10-AB (marked by open circle in **Figure 1B**; [11]). In addition, the position of a second methionine preceding the one above in XFIN-B is usually occupied by a basic residue (arginine or lysine) in most deduced frog KRAB-B subdomains. Secondary structure predictions for the ZNF10 and XFIN KRAB-AB domains using public webservers did not give any clues to explain functional differences (data not shown). Structural investigations suggested that KRAB-A as well as KRAB-AB domains in general lack a stable structure and can be considered unfolded conformers with residual secondary structure that fold upon binding to their interaction partner TRIM28 ([18], [71]). The only available structural data of a KRAB domain in the Protein Data Bank (PDB) originated from unpublished nuclear magnetic resonance experiments (PDB ID 1V65; Saito K, Koshiba S, Inoue M., Kigawa T., Yokoyama S. Solution structure of the Kruppel-associated box (KRAB) domain). The structure of the respective investigated mouse KRAB, namely a KRAB-A followed by a weak KRAB-C subdomain, proposes two alpha-helical stretches around the most conserved residues of KRAB-A.

Reports on biochemical characteristics of the TRIM28/KRAB-AB interaction are limited. The stoichiometry was shown to be a 3∶1 ratio of TRIM28 over KRAB [18]. The only kinetic data of this interaction we are aware off were determined for the TRIM28/ZNF10-AB interaction and reported a dissociation constant of 142 nM [72]. Such investigations appear to be hindered by the poor efficiency of TRIM28 and KRAB proteins in forming complexes under test tube conditions. Efficient interaction appears to require *in vivo* conditions or at least *in vitro* co-translation [18], [73]. Altogether, more biochemical and structural experiments in conjunction with molecular modeling are necessary to advance our understanding of the KRAB/TRIM28 module.

The fact that repression was observed in *Xenopus laevis* cells for both, XFIN and ZNF10 KRAB domains, suggested that already the common ancestor of amphibians and mammals contained a functional KRAB/TRIM28 module. We observed significant weaker overall repression factors for both tested KRAB domains in frog compared to human cells, but no repression in fish cells. Transcriptional repression in frog cells could be reproduced with a different *Xenopus laevis* frog cell line (XTC-2 cells, data not shown). It is tempting to speculate that the disparate transcriptional repression observed in both cellular systems could be due to fine-tuning or enhancements of factors in the mammalian lineage that have been evolved during tetrapode evolution. In addition, it will be interesting to investigate whether any other *Xenopus* KRAB domain might be more potent in conferring transcriptional repression in amphibian cells. Based on current gene models in the frog databases, there are KRAB-A only as well as KRAB-AB proteins. Compared to XFIN, several *Xenopus* proteins containing KRAB-A only as well as KRAB-B domains from AB configurations display higher HMM scores (against human as well as amphibian KRAB-A and –B matrices; see **Table S2**). Yet, according to these scores XFIN-AB appears to be in the upper ranks of all frog KRAB domains and a fair representative for functional tests.

**Figure 7:**
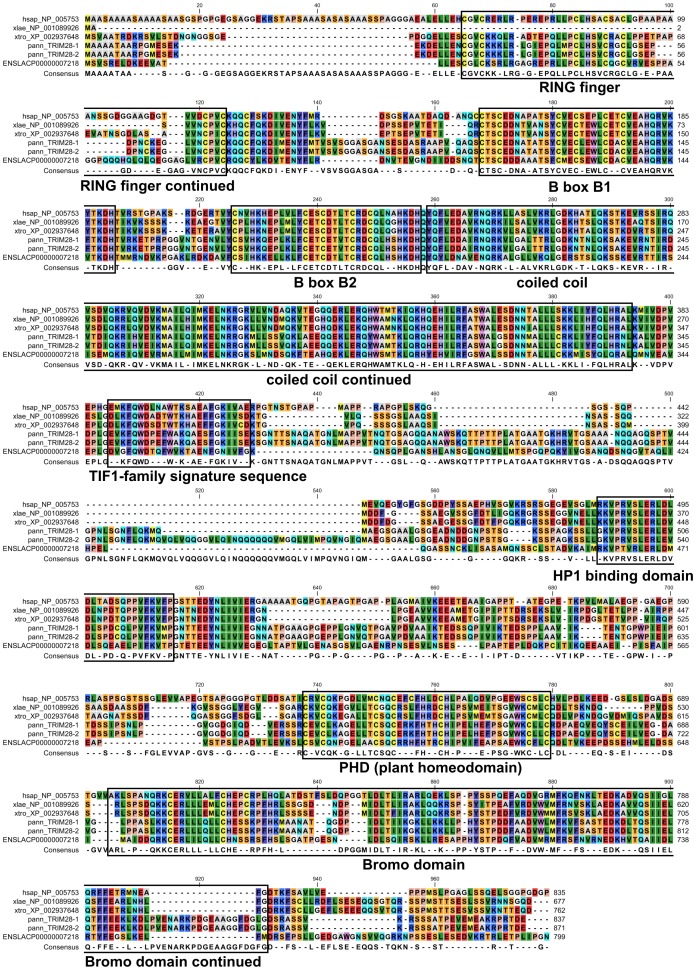
Protein alignments of human TRIM28 with the putative orthologs from *Xenopus* and lobe-finned fish species. Sequences are designated according to their database accession numbers (NCBI Refseq or ENSEMBL) where available and a species prefix (hsap = *Homo sapiens*; xlae = *Xenopus laevis*; xtro = *Xenopus tropicalis*; pann = *Protopterus annectens* (lungfish); lcha = *Latimeria chalumnae* (coelacanth)). The lungfish sequences do not bear official identifiers yet and are named arbitrarily here. They were obtained as described in Materials and Methods (Bioinformatics section). The original transcript sequences are provided in **Table S2**. The consensus sequence under the alignments is based on the occurence of the shown amino acids in at least 60% of the molecules. Reciprocal BLASTp of the frog and fish sequences against human sequence databases resulted in human TRIM28 as the best hit and thus supported the ortholog assignment (data not shown). Boxes around and labels under the sequence alignments delineate the domain organization based on human TRIM28 [80].

In databases, XFIN is often also called ZNF208. Indeed, when interrogating the human proteins by BLASTp with *Xenopus laevis* XFIN, human ZNF208 (Refseq NP_009084) has the highest score. However, reciprocal BLASTp in *Xenopus* resulted in other ZNF entries as top hits that do not carry KRAB domains (data not shown). The ortholog search based on sequence homology over the large phylogenetic gap between mammals and frog is hindered by the many highly conserved zinc finger sequences. A *bona fide* ortholog for XFIN based on sequence similarity could only be determined in *Xenopus tropicalis*. It is highly likely that the genome sequences encoding the NCBI XP_002942031 protein (also included in **Table S2**) contain the proper XFIN ortholog although the exact gene model resulting in this predicted protein might be preliminary. TRIM28 in *Xenopus laevis* is not well characterized. We did a BLASTp database search with human TRIM28 and chose the best hit each in *Xenopus laevis* (NP_001089926) and *Xenopus tropicalis* (NCBI XP_002937648), respectively. Reciprocal BLASTp against all human protein sequences defined TRIM28 as the human protein with highest homology and thus confirmed the two candidates as the frog orthologs. These results fit the entries in the Xenbase *Xenopus* database (http://www.xenbase.org). The alignments of the putative Xenopus TRIM28 proteins to human TRIM28 illustrate their conserved domain organization (**Figure 7**). The likely existence of amphibian TRIM28 proteins is an additional support for the above statement of a functional KRAB/TRIM28 module in the oldest class of tetrapodes.

According to the few available studies, XFIN appears to be a cytoplasmic protein with a higher affinity towards RNA [40], [41]. The KRAB protein-protein interaction domain is usually considered to be associated with pathways of transcriptional repression in the nucleus, as reviewed in the introduction and discussed above. This raises the question what functions XFIN might mediate in the cytoplasm in the context of RNA metabolism. Clear-cut biological roles of KRAB-ZNF proteins in the cytoplasm have not been elucidated so far. Yet, association of other KRAB-ZNF proteins to RNA (human ZNF74, [66]), association with snRNPs (ZFP100, synonym ZNF473; [74]) and ribosomes (ZNF7; [75]) have been reported. Further, the existence of cytoplasmic pools of KRAB-ZNF proteins that translocate into the nucleus under particular conditions has also been documented (PARIS/ZNF746, [23]; NRIF/Zfp110, [76]).

In numerous publications, the KRAB domain has been called tetrapode-specific based on sequence homologies and the derived assumption that KRAB domains encoded by *Xenopus* genes should mediate repressor activity. Yet, up to now, no investigations on KRAB-mediated transcriptional repression in fish or on *Xenopus* KRAB domains have been published. The origin of the KRAB domain has been recently challenged by the discovery of the Meisetz/PRDM9 ortholog group, for which members can clearly be defined in all vertebrate classes and even in at least an invertebrate, in the sea urchin [8]. However, here we show that the human progeny of this postulated putative predecessor of the KRAB domain does not confer any transcriptional repressor activity in human HeLa cells despite strong sequence homologies to well-known human KRAB domains. This result could be explained by the deviation from the consensus KRAB-A sequence matrix at certain positions (labeled in **Figure 1A**) that resulted in only moderate E-values against HMM profiles of human KRAB-A. Particular examples include the methionine (position 20 of the alignment) instead of the usual leucine, a basic lysine (position 32 of the alignment) in lieu of methionine and the missing acidic residue (position 34 of the alignment) resulting in a gap in the alignment. In agreement with database annotations, a comparison against KRAB-B HMMs did not show any evidence for KRAB-B-like amino acid sequences in PRDM9. However, the absence of a B subdomain as such does not exclude potent transcriptional repression activity, since KRAB-A subdomains from KRAB-zinc finger proteins that only exhibit them alone were shown to confer distinct repression [28], [35], [77].

## Conclusions

We propose to define those KRAB domains as functional that show significant transcriptional repression activity in reporter assays and are able to interact with TRIM28. Based on the current state of knowledge, thus, functional KRAB domains can only be found in tetrapodes and homologs in non-tetrapodes might be considered the progeny of KRAB-like ancestor domains. The joint lack of both, functional KRAB domains and TRIM28, in ray-finned fish species are consistent with the concept that a functional KRAB/TRIM28 system of transcriptional modulation evolved in the common ancestor of the tetrapodes. Interestingly, sequences from two lobe-finned fish species, namely the recently sequenced genome of the coelacanth *Latimeria chalumnae* and transcripts obtained from lungfish (*Protopterus annectens*) tissues [58], apparently contain putative TRIM28 orthologs (**Figure 7**) and proteins with KRAB-like sequences (e.g. ENSLACP00000004712). These findings argue that the *bona fide* functional KRAB/TRIM28 module might have started to evolve around the split between lobe-finned fish and tetrapodes.

## Supporting Information

Figure S1 **Western blot analysis to compare the expression of the various Gal4 fusion protein effector constructs.** Total protein extracts were made with SDS sample buffer and equal volumes of the extracts from within one experiment were subjected to Western blotting. **A, B**: Example blots for expression in human HeLa cells for the indicated constructs (Gal4 alone or Gal4-KRAB fusions). Blots were separated in two molecular weight portions and stained with anti-GAPDH for normalization (a) or anti-Gal4 (b) to visualize the Gal4 fusion proteins. The average relative normalized Gal4/GAPDH expression values (+/− standard deviation SD) were calculated from four independent experiments using the fluorescence signals measured by the LI-COR Odyssey® fluorescence imager software. **C**: Experiment in *Xenopus laevis* A6 cells. As in A, B, with the exception that beta-actin was used for normalization purposes. * Statistical evaluation (paired 2-tailed T-test) compared the ZNF10-AB construct to the other KRAB constructs only.
(PDF)

Figure S2 **Western blot analysis to look at the level of expression for the various Gal4 fusion proteins of the N-terminal PRDM9 portion.** Total protein extracts were made with SDS sample buffer and equal volumes of the extracts. Experimental workflow and execution as described in Figure S1.
(PDF)

Figure S3 **Distribution of ectopically expressed Gal4-PRDM9 N-terminal fusion proteins and colocalization analysis with endogenous TRIM28 in human HeLa cells.** Cells were fixed and stained 24 hours after transfection. Each row shows the cells of the same image pane. Note, that there were no clear-cut nucleoplasmic foci in which PRDM9 domains and endogenous TRIM28 were both enriched. Bar = 10 µm.
(PDF)

Figure S4 **Intracellular distribution of Gal4 and Gal4-NES KRAB fusion proteins 48 hours after transfection with the indicated constructs.** Staining with anti-Gal4 antibodies **A**: Visualization by indirect immunofluorescence microscopy in *Xenopus laevis* A6 cells. Individual image panes a-h. Bar = 15 µm. **B**: Expression in *Pimephales promelas* EPC cells. Individual image panes a–e. Bar = 10 µm.
(PDF)

Table S1 **DNA oligonucelotides used to construct plasmids described in the manuscript.**
(XLS)

Table S2 **List of sequences used in bioinformatic analyses.** Part A: List of the presumable frog KRAB domain proteins that were used to build the HMMs of frog KRAB-A and -B. Part B: Putative TRIM28 ortholog transcripts from lungfish (*Protopterus annectens*).
(XLS)

Table S3 **Quantitative data from the manual scoring and counting of the compartmentalization assay of the Gal4-NES-KRAB constructs in human HeLa, frog** ***Xenopus laevis*** **A6 and fish** ***Pimephales promelas*** **EPC cells.**
(XLS)
