# Supplementary material for: The B-Subdomain of the Xenopus laevis XFIN KRAB-AB Domain Is Responsible for Its Weaker Transcriptional Repressor Activity Compared to Human ZNF10/Kox1
Source: PLoS One. 2014 Feb 3;9(2):e87609. doi: 10.1371/journal.pone.0087609 (PMC3912051; doi:10.1371/journal.pone.0087609)
Supplement: Figure S1 — Western blot analysis to compare the expression of the various Gal4 fusion protein effector constructs. Total protein extracts were made with SDS sample buffer and equal volumes of the extracts from within one experiment were subjected to Western blotting. A, B: Example blots for expression in human HeLa cells for the indicated constructs (Gal4 alone or Gal4-KRAB fusions). Blots were separated in two molecular weight portions and stained with anti-GAPDH for normalization (a) or anti-Gal4 (b) to visualize the Gal4 fusion proteins. The average relative normalized Gal4/GAPDH expression values (+/− standard deviation SD) were calculated from four independent experiments using the fluorescence signals measured by the LI-COR Odyssey® fluorescence imager software. C: Experiment in Xenopus laevis A6 cells. As in A, B, with the exception that beta-actin was used for normalization purposes. * Statistical evaluation (paired 2-tailed T-test) compared the ZNF10-AB construct to the other KRAB constructs only. (PDF) [file pone.0087609.s001.pdf]

A

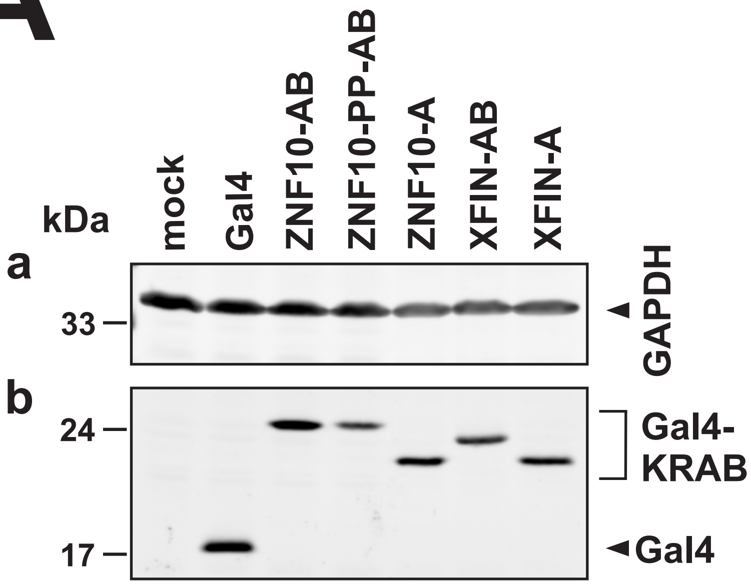

c

Relative normalized expression

Construct	Mean	SD	p-value *
Gal4	1		
ZNF10-AB	0.78	0.27	
ZNF10-PP-AB	0.37	0.13	0.02
ZNF10-A	0.78	0.26	0.94
XFIN-AB	0.63	0.18	0.10
XFIN-A	0.66	0.16	0.35

n = 4

B

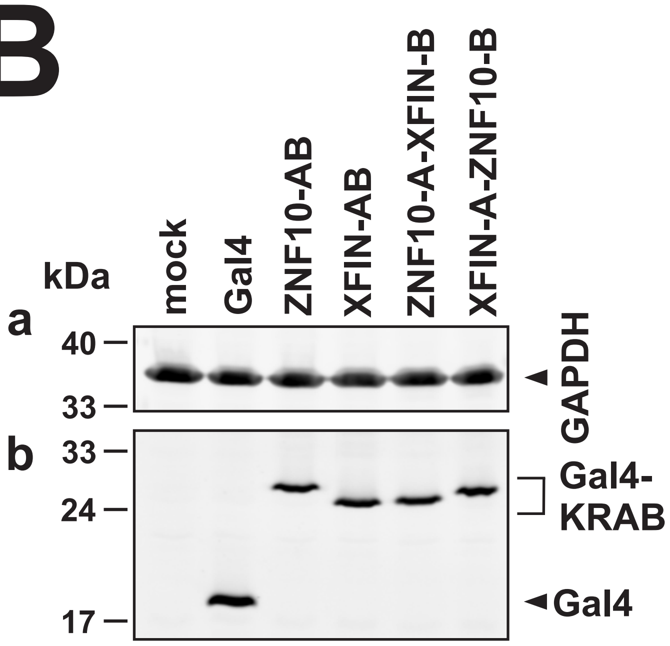

c

Relative normalized expression

Construct	Mean	SD	p-value *
Gal4	1	0	
ZNF10-AB	0.61	0.14	
XFIN-AB	0.56	0.09	0.57
ZNF10-A-XFIN-B	0.48	0.09	0.17
XFIN-A-ZNF10-B	0.52	0.15	0.25

n = 4

C

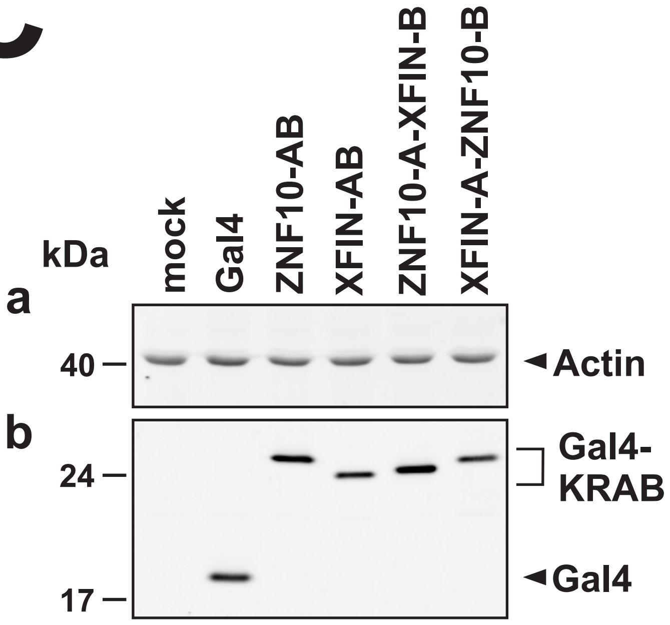

c

Relative normalized expression

Construct	Mean	SD	p-value *
Gal4	1	0	
ZNF10-AB	1.13	0.23	
XFIN-AB	0.61	0.22	0.001
ZNF10-A-XFIN-B	1.40	0.33	0.030
XFIN-A-ZNF10-B	0.61	0.20	0.005

n = 4
