# Supplementary material for: The B-Subdomain of the Xenopus laevis XFIN KRAB-AB Domain Is Responsible for Its Weaker Transcriptional Repressor Activity Compared to Human ZNF10/Kox1
Source: PLoS One. 2014 Feb 3;9(2):e87609. doi: 10.1371/journal.pone.0087609 (PMC3912051; doi:10.1371/journal.pone.0087609)
Supplement: Figure S2 — Western blot analysis to look at the level of expression for the various Gal4 fusion proteins of the N-terminal PRDM9 portion. Total protein extracts were made with SDS sample buffer and equal volumes of the extracts. Experimental workflow and execution as described in Figure S1. (PDF) [file pone.0087609.s002.pdf]

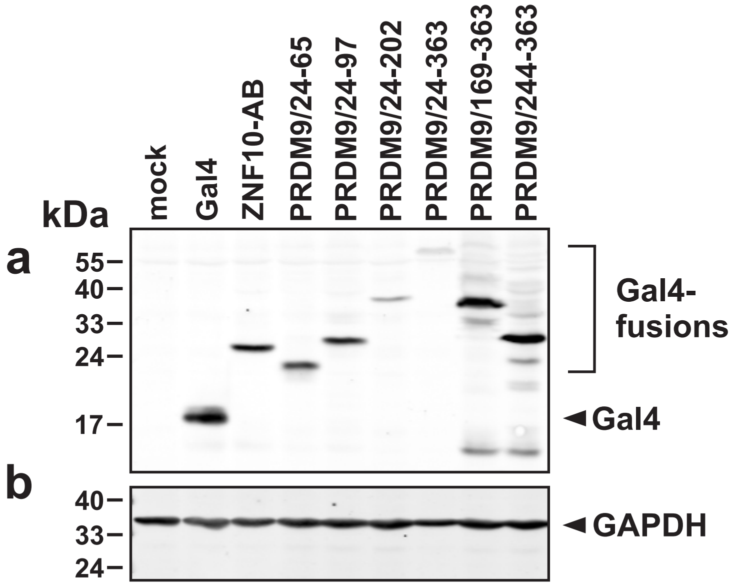

c

Relative normalized expression

Construct	Mean	SD	p-value *
Gal4	1.00	0.00	
ZNF10-AB	0.75	0.24	
PRDM9/24-65	0.40	0.07	0.08
PRDM9/24-97	0.35	0.15	0.12
PRDM9/24-202	0.21	0.11	0.03
PRDM9/24-363	0.15	0.13	0.02
PRDM9/169-363	0.67	0.26	0.68
PRDM9/244-363	0.78	0.12	0.89

n = 4
