# Supplementary figures and images for: The B-Subdomain of the Xenopus laevis XFIN KRAB-AB Domain Is Responsible for Its Weaker Transcriptional Repressor Activity Compared to Human ZNF10/Kox1
Source: PLoS One. 2014 Feb 3;9(2):e87609. doi: 10.1371/journal.pone.0087609 (PMC3912051; doi:10.1371/journal.pone.0087609)

# HeLa (human)

Gal4

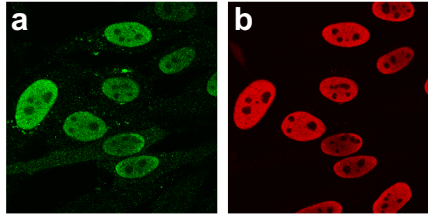

PRDM9/  
24-65

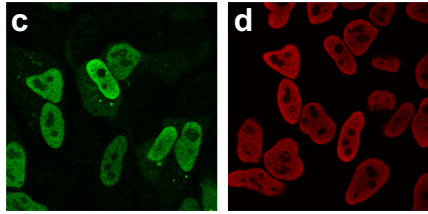

PRDM9/  
24-97

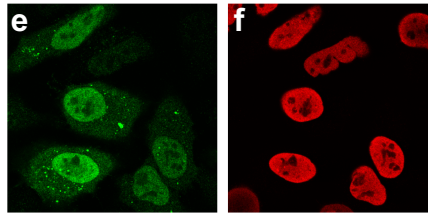

PRDM9/  
24-202

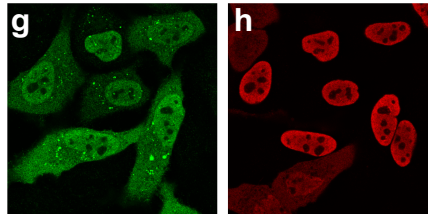

PRDM9/  
24-363

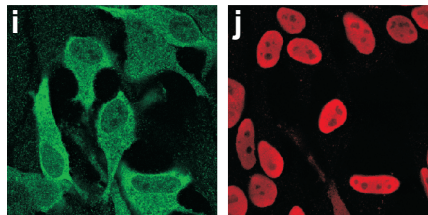

PRDM9/  
169-363

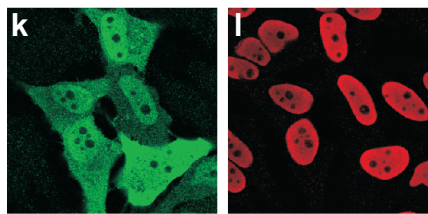

PRDM9/  
244-363

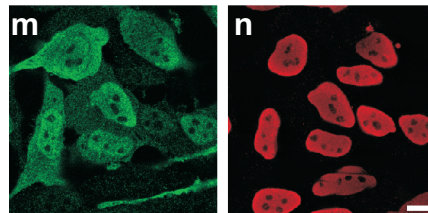

anti-Gal4

anti-TRIM28

Supplement: Figure S3 — Distribution of ectopically expressed Gal4-PRDM9 N-terminal fusion proteins and colocalization analysis with endogenous TRIM28 in human HeLa cells. Cells were fixed and stained 24 hours after transfection. Each row shows the cells of the same image pane. Note, that there were no clear-cut nucleoplasmic foci in which PRDM9 domains and endogenous TRIM28 were both enriched. Bar = 10 µm. (PDF) [file pone.0087609.s003.pdf]

**A**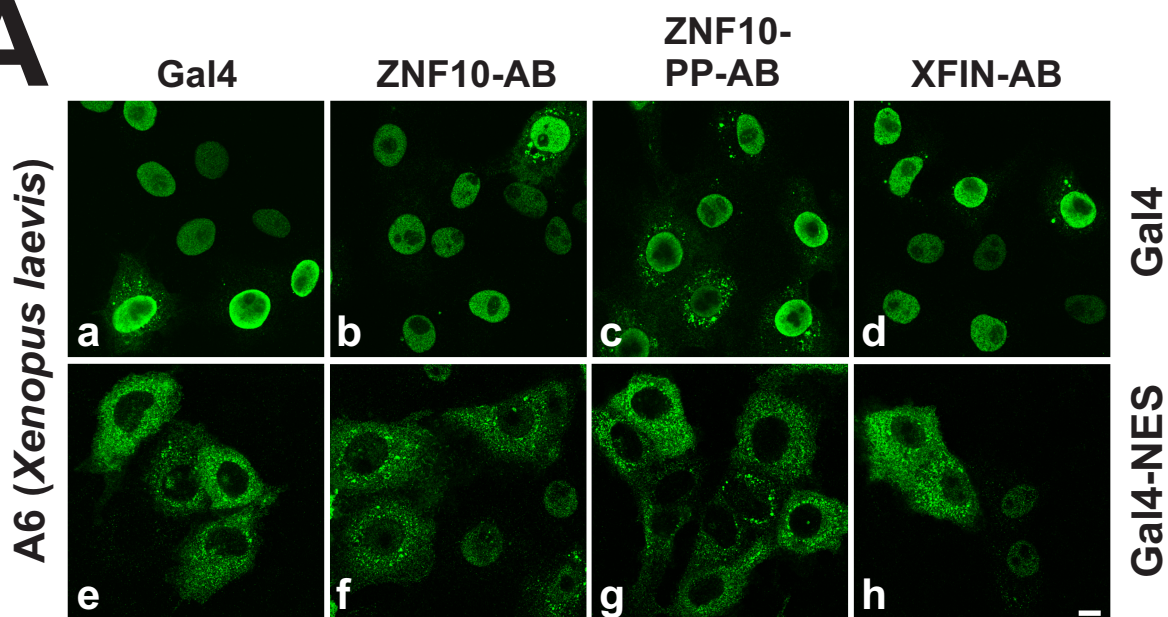**B**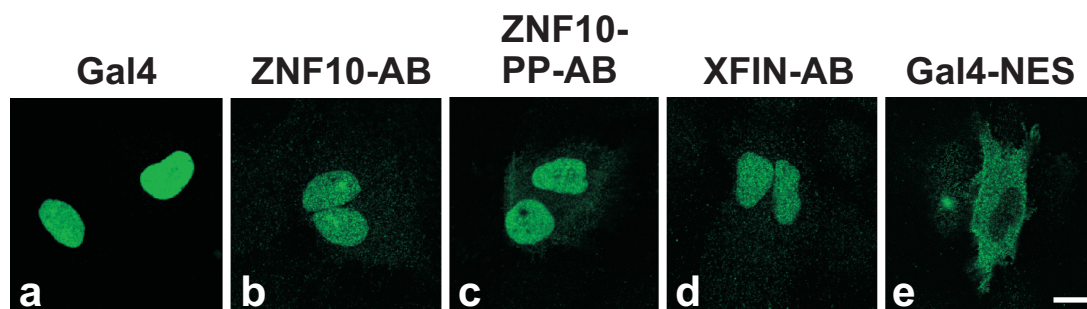

EPC (*Pimephales promelas*)

Supplement: Figure S4 — Intracellular distribution of Gal4 and Gal4-NES KRAB fusion proteins 48 hours after transfection with the indicated constructs. Staining with anti-Gal4 antibodies A: Visualization by indirect immunofluorescence microscopy in Xenopus laevis A6 cells. Individual image panes a-h. Bar = 15 µm. B: Expression in Pimephales promelas EPC cells. Individual image panes a–e. Bar = 10 µm. (PDF) [file pone.0087609.s004.pdf]
